# Functional imaging of cortical feedback projections to the olfactory bulb

**DOI:** 10.3389/fncir.2014.00073

**Published:** 2014-07-03

**Authors:** Markus Rothermel, Matt Wachowiak

**Affiliations:** Brain Institute and Department of Neurobiology and Anatomy, University of UtahSalt Lake City, UT, USA

**Keywords:** olfactory bulb, centrifugal systems, anterior olfactory nucleus, GCaMP, *in vivo*

## Abstract

Processing of sensory information is substantially shaped by centrifugal, or feedback, projections from higher cortical areas, yet the functional properties of these projections are poorly characterized. Here, we used genetically-encoded calcium sensors (GCaMPs) to functionally image activation of centrifugal projections targeting the olfactory bulb (OB). The OB receives massive centrifugal input from cortical areas but there has been as yet no characterization of their activity *in vivo*. We focused on projections to the OB from the anterior olfactory nucleus (AON), a major source of cortical feedback to the OB. We expressed GCaMP selectively in AON projection neurons using a mouse line expressing Cre recombinase (Cre) in these neurons and Cre-dependent viral vectors injected into AON, allowing us to image GCaMP fluorescence signals from their axon terminals in the OB. Electrical stimulation of AON evoked large fluorescence signals that could be imaged from the dorsal OB surface *in vivo*. Surprisingly, odorants also evoked large signals that were transient and coupled to odorant inhalation both in the anesthetized and awake mouse, suggesting that feedback from AON to the OB is rapid and robust across different brain states. The strength of AON feedback signals increased during wakefulness, suggesting a state-dependent modulation of cortical feedback to the OB. Two-photon GCaMP imaging revealed that different odorants activated different subsets of centrifugal AON axons and could elicit both excitation and suppression in different axons, indicating a surprising richness in the representation of odor information by cortical feedback to the OB. Finally, we found that activating neuromodulatory centers such as basal forebrain drove AON inputs to the OB independent of odorant stimulation. Our results point to the AON as a multifunctional cortical area that provides ongoing feedback to the OB and also serves as a descending relay for other neuromodulatory systems.

## Introduction

Sensory systems enable an animal to detect and act upon relevant environmental information in order to navigate and survive in a complex world. Sensation is an active process in which external stimuli are selectively sampled in space and time, and the processing of incoming sensory information is strongly and dynamically modulated depending on behavioral state and past experience. Thus, activity at all stages of sensory pathways is not solely determined by sensory input but also by ongoing activity in other brain areas. Understanding the neural mechanisms underlying sensation thus requires understanding the neural circuits mediating this modulation.

Similar to other systems such as the visual system where behavioral state modulates early sensory processing (Niell and Stryker, 2010; Fu et al., 2014), response properties of neurons in the early olfactory pathway are modulated in the behaving animal. Numerous studies have investigated the modulation of activity in the olfactory bulb (OB)—the first stage of synaptic processing of olfactory sensory input—as a function of behavioral state and found rapid and profound effects (Karpov, 1980; Kay and Laurent, 1999; Doucette and Restrepo, 2008; Kato et al., 2012; Wachowiak et al., 2013; Nunez-Parra et al., 2014). It has been hypothesized that centrifugal modulation from diverse brain centers plays an important role in mediating these effects (Matsutani and Yamamoto, 2008; Shea et al., 2008; Petzold et al., 2009; Nunez-Parra et al., 2013; Rothermel et al., 2014). The OB receives centrifugal input from fibers originating in classical neuromodulatory centers including noradrenergic inputs from locus coeruleus (Shipley et al., 1985; McLean et al., 1989; Shea et al., 2008), serotonergic inputs from raphe (Mclean and Shipley, 1987; Petzold et al., 2009) and cholinergic and GABA-ergic inputs from the basal forebrain (Ichikawa and Hirata, 1986; Ojima et al., 1988; Nunez-Parra et al., 2013; Rothermel et al., 2014). The OB is also heavily innervated by centrifugal projections originating throughout olfactory cortex (Price and Powell, 1970; Davis et al., 1978; de Olmos et al., 1978; Haberly and Price, 1978; Reyher et al., 1988; De Carlos et al., 1989; Matsutani, 2010). Despite extensive characterization of these projections, the functional properties of centrifugal input to the OB *in vivo* has yet to be described for any system.

The anterior olfactory nucleus (AON) constitutes the largest source of OB centrifugal inputs to the OB (Carson, 1984; Shipley and Adamek, 1984). It is the most anterior subdivision of olfactory cortex and can be divided into two distinct zones: pars externa, consisting of a thin ring of cells surrounding the rostral end of the AON, and the remainder, pars principalis which itself can be further subdivided into 4 parts (dorsal, lateral, medial and ventral) (Valverde et al., 1989; Brunjes et al., 2005). The AON receives sensory input from the OB and sends “ascending” outputs to other olfactory and non-olfactory areas including anterior piriform cortex, olfactory tubercle, entorhinal cortex and periamygdaloid cortex (for review, see Brunjes et al., 2005). Features of the connections between the AON and the OB include a coarse topography in the centripetal projections from the OB to the AON (Schoenfeld et al., 1985; Scott et al., 1985; Yan et al., 2008; Miyamichi et al., 2011) as well as descending projections that innervate not only the ipsilateral but also the contralateral OB (Schoenfeld and Macrides, 1984; Shipley and Adamek, 1984; Kay and Brunjes, 2014). In addition, laminar differences in the distribution of AON projections to OB have been observed for both zones (Davis and Macrides, 1981; Luskin and Price, 1983). Finally, the AON itself receives robust centrifugal inputs from other olfactory cortical areas including anterior piriform cortex (Haberly and Price, 1978; Luskin and Price, 1983; Haberly, 2001) and amygdala (De Carlos et al., 1989; Gomez and Newman, 1992; Canteras et al., 1995; Petrovich et al., 1996) as well as higher-order centers such as basal forebrain (Broadwell and Jacobowitz, 1976; Luiten et al., 1987; De Carlos et al., 1989; Carnes et al., 1990; Gaykema et al., 1990; Zaborszky et al., 2012) and the hippocampus (Swanson and Cowan, 1977; van Groen and Wyss, 1990). This extensive connectivity with primary and secondary olfactory processing centers and its position as both a relay of ascending sensory input from the OB and a source of “top-down”, centrifugal input to the OB makes this structure an interesting model system for investigating higher-order olfactory processing.

The AON has been implicated in a range of different functions in odor perception, including serving as the first site of integrated odor percept formation, reconstructing olfactory memory traces (Haberly, 2001), and integrating activity within and between the two OBs (Schoenfeld and Macrides, 1984; Lei et al., 2006; Kikuta et al., 2010). However, the role of centrifugal AON projections in modulating ongoing OB activity remains poorly characterized, especially in a functional and behavioral context. So far, only one study has investigated the influence of centrifugal AON projections on OB circuit function (Markopoulos et al., 2012); this study demonstrated that optogenetically activating these inputs directly depolarizes as well as disynaptically inhibits mitral/tufted cells, thereby enabling precisely timed spikes in a population of mitral/tufted cells and shaping OB output. However, how centrifugal AON fibers are activated naturally remains unclear.

In the present study, we used genetically-encoded calcium reporters (GCaMPs) to functionally image the activation of AON projections innervating the OB in the anesthetized and awake mouse. We found that olfactory sensory input rapidly and robustly activates AON feedback projections to the ipsi- as well as contralateral OB which are transient and coupled to inhalation in both the anesthetized and the awake animal. AON feedback projections could also be activated by higher-order neuromodulatory centers. Two-photon imaging revealed distinct spatiotemporal patterns of AON feedback evoked by different odorants. These results provide the first *in vivo* functional characterization of centrifugal inputs to the OB, and point to the AON as an integral olfactory processing center that provides robust, ongoing and odorant-specific feedback to the OB and also serves as a relay for other neuromodulatory systems.

## Materials and methods

### Animals strain and care

We used a mouse line (Chrna7-Cre, kindly provided by S. Rogers and P. Tvrdik, University of Utah) in which an IRES-Cre bi-cistronic cassette was introduced into the 3’noncoding region of the cholinergic nicotinic receptor alpha7 (*Chrna7*) (Rogers and Gahring, 2012; Rogers et al., 2012a,b; Gahring et al., 2013). Additional experiments were performed on mice expressing Cre recombinase (Cre) under the olfactory marker protein promotor (OMP; Li et al., 2004), JAX Stock #006668 (The Jackson Laboratory) crossed to the Ai38 reporter line (Zariwala et al., 2012), JAX Stock #014538 (The Jackson Laboratory). Animals of either sex were used. Animals were housed under standard conditions in ventilated racks. Mouse colonies were bred and maintained at the University of Utah animal care facilities. All procedures were carried out following National Institutes of Health Guide for the Care and Use of Laboratory Animals and were approved by the University of Utah Institutional Animal Care and Use Committee.

### Viral vectors

Viral vectors were obtained from the viral vector core of the University of Pennsylvania. Vectors were from stock batches available for general distribution. Injection of Cre-dependent vector was performed in either heterozygous or homozygous Chrna7-Cre mice. Virus injection was targeted to the dorsal OB at a depth of 200–300 μm or to the AON using stereotaxic targeting (relative to Bregma (in mm) +2.8 anteroposterior, 1.25 mediolateral, −2.6 dorsoventral) using previously-described procedures (Wachowiak et al., 2013). Virus (0.1–0.2 μl for OB injections; 0.5 μl for AON injections) was delivered through a 33 or 30 gauge metal needle (AON injections) or a pulled glass pipette (OB injections) at a rate of 0.1 μl/min. Mice were between 4 and 12 weeks of age at the time of virus injection and were individually housed for 14–28 days before evaluating for transgene expression or imaging. In a few cases (see Section Results), we injected virus into the OB of postnatal pups (P 1–3) and evaluated expression at 6–16 weeks of age. The viral vectors used, with their abbreviated names as used in the text, were: AAV2/1.hSynap.FLEX.GCaMP3.3.WPRE.SV40 (*2/1.FLEX.GCaMP3*) and AAV2/9.Syn.Flex.GCaMP6s.WPRE.SV40 (*2/9.FLEX.GCaMP6s*).

### Olfactometry

Odorants were presented as dilutions from saturated vapor in cleaned, humidified air using a custom olfactometer under computer control (Bozza et al., 2004; Verhagen et al., 2007). Odorants were typically presented for 4 s. All odorants were obtained at 95–99% purity from Sigma-Alrich and stored under nitrogen. The concentration of the odorants ranged from 0.1 to 2% saturated vapor (s.v.).

### Epifluorescence imaging

For acute imaging experiments, mice were anesthetized with pentobarbital (50 mg/kg). Body temperature and heart rate were maintained at 37°C and ∼400 beats per minute. Unless otherwise stated, a double tracheotomy was performed and an artificial inhalation paradigm used (Wachowiak and Cohen, 2001; Spors et al., 2006). Animals were secured in a stereotaxic device (Kopf Instrument) for further procedures and imaging followed previously established protocols (Wachowiak and Cohen, 2001; Bozza et al., 2004; Spors et al., 2006). Imaging in awake, head-fixed mice was performed using an identical optical setup that accommodated a custom restraint and behavioral training apparatus (described below). Wide-field epifluorescence signals were acquired through the thinned bone overlying the dorsal OB. The optical setup included an Olympus BX51 illumination turret, an Olympus 4x 0.28 numerical aperture objective, a filter set optimized for GFP (exciter: HQ480/40, dichroic: Q505LP, emitter: HQ535/50, Semrock), and a 470 nm light-emitting diode (LED) source (Thorlabs) or 150W Xenon arc lamp (Opti-quip). Light was attenuated using neutral density filters. Optical signals were acquired at 256 × 256 pixel resolution and a frame rate of 25 Hz and digitized at 14-bit resolution using a back-illuminated charge-coupled device (CCD) camera (NeuroCCD, SM-256, RedShirtImaging), then synchronized with other experimental signals (respiration, odor control) and stored to disk using Neuroplex software (RedShirtImaging).

### Electrical stimulation

Electrical stimulation of AON or horizontal limb of the diagonal band of Broca (HDB) was performed using a concentric bipolar electrode (CBCPH-75, FHC) inserted through a small craniotomy to the same stereotaxic coordinates used for virus injection or, in the case of HDB stimulation, to coordinates (relative to Bregma (in mm) +0.74 anteroposterior, 0.65 mediolateral, −4.8 dorsoventral. Stimulus trains were composed of 300 μA, 300 μs duration pulses delivered at 50 Hz for 0.1–1 s, as specified in the text.

### Awake, head-fixed preparation

Epifluorescence imaging in awake, head-fixed mice was performed as described previously (Wachowiak et al., 2013). Mice were acclimated to head restraint for 1–2 daily sessions prior to imaging, with no operant conditioning. Persistent limb movement or severely attenuated respiration was used as an indicator of excessive stress, in which case the session was terminated. A single imaging session lasted for up to 60 min and data were collected over as many as two consecutive daily sessions. To compare optical signals during wakefulness and anesthesia (Figure 4), head-fixed mice were briefly anesthetized with isoflurane, then responses to odorants imaged in the 1–2 min after anesthetic was removed (Wachowiak et al., 2013).

**Figure 1:**
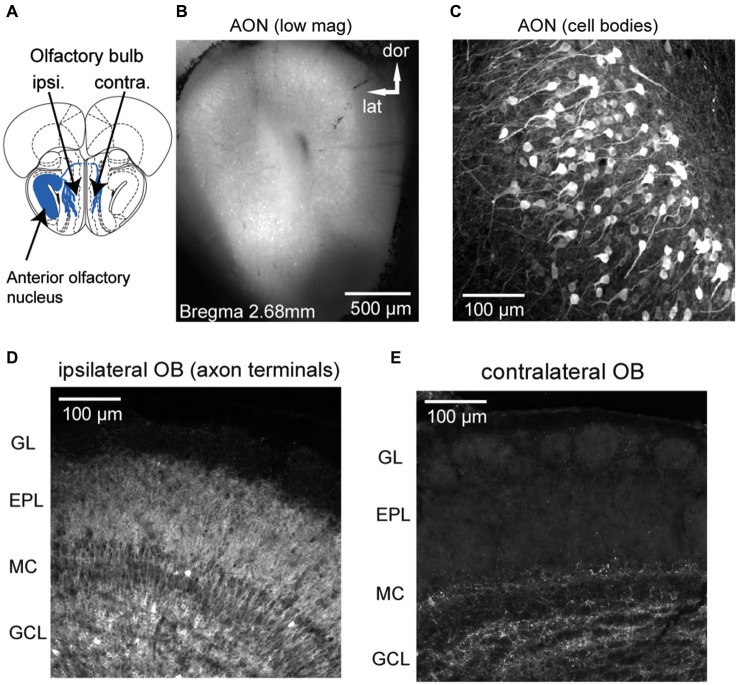
**GCaMP3 expression after unilateral viral injection into AON of a Chrna7-Cre mouse. (A)** Schematic diagram (Bregma 3.08 mm, section from atlas (Paxinos and Franklin, 2001)) depicting genetically-targeted AON projections to the ipsilateral and contralateral OB. **(B)** Low-magnification, epifluorescence image showing heavy expression in AON neurons in all AON subdivisions. **(C)** Confocal stack showing GCaMP3 expression in AON cell bodies and neurites. Note thick apical dendrites and “pyramidal-type” morphology of expressing neurons.** (D and E)** Confocal stack from the ipsilateral **(D)** and contralateral **(E)** dorsal OB displaying AON terminals ending in different layers. The ipsilateral OB shows expression predominately in the granule cell and external plexiform layers whereas in the contralateral OB fewer fibers in granule cell and deep external plexiform layer are labeled. GL: glomerular layer, EPL: external plexiform layer, MC: mitral cell layer, GCL: granule cell layer.

**Figure 2:**
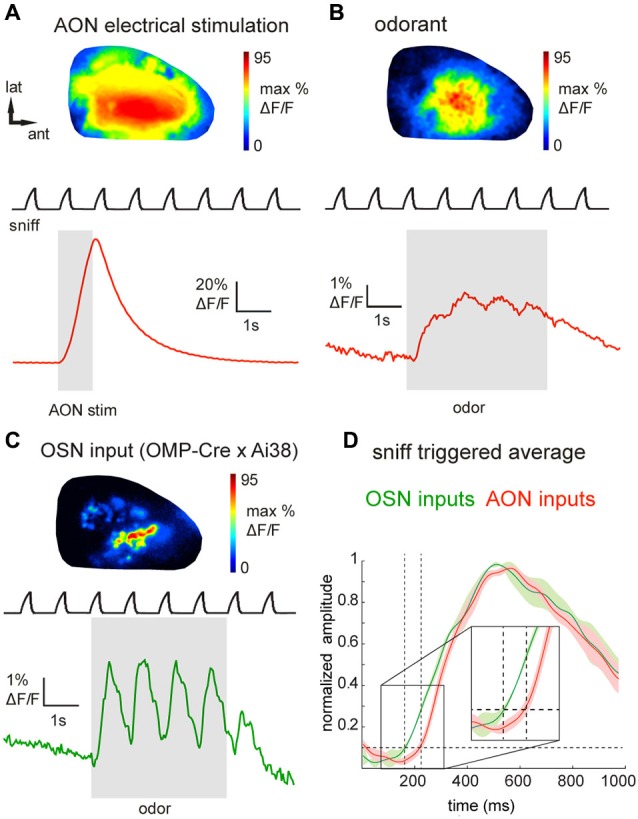
**Epifluorescence imaging of centrifugal and olfactory sensory neuron inputs to the OB *in vivo* using GCaMP3. (A)** GCaMP3 signals imaged from the dorsal OB of an anesthetized Chrna7-Cre mouse after viral injection into ipsilateral AON. Top: Map of response to electrical stimulation of AON showing widespread fluorescence increases. Map is normalized to its own maximum. Bottom: Time-course of the optical signal. AON stimulation causes a large, transient increase in GCaMP3 fluorescence that decays rapidly. Upper trace (“sniff”) shows intranasal pressure transients during artificial inhalation. **(B)** Map (top) and time-course (bottom) of odorant-evoked (1% hexanal) GCaMP3 signals, displayed as in **(A)**. Odorant presentation evokes large fluorescence changes that are driven by inhalation and broadly distributed across the dorsal OB. **(C)** Map (top) and time-course (bottom) of odorant-evoked (1% ethyl butyrate) GCaMP3 signals imaged from OSN axonal terminals in an anesthetized OMP-Cre:Ai38 mouse. Signals appear as discrete, glomerular foci and display strongly-modulated inhalation-linked response dynamics. **(D)** “Sniff-triggered” average GCaMP3 signals comparing responses from OMP-Cre:Ai38 animals (green trace) to Chrna7-Cre animals expressing GCaMP3 in the AON (red trace). Both traces are normalized to their own maxima. Traces show mean ± SEM of the inhalation-aligned response. Sensory neuron signals display a faster onset (see also insert) compared to the signals imaged in the Chrna7-Cre animals.

**Figure 3:**
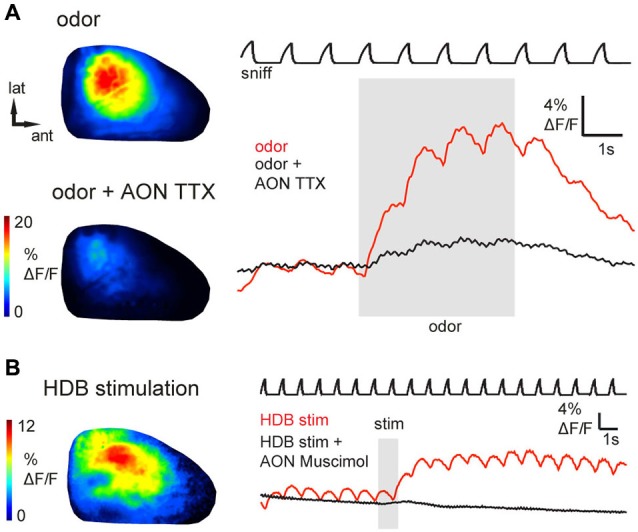
**Epifluorescence signals arise from AON activity and can be activated by basal forebrain stimulation. (A)** Odorant-evoked (1% ethyl butyrate) GCaMP6s signals before and after injection of TTX into AON (see Text for details). Shown are odorant response maps (left) and optical signal traces (right) taken immediately before (upper map, red trace) or after (lower map, black trace) TTX injection. TTX injection into the AON reduced odorant-evoked responses in the OB. **(B)** Response maps and time-course of GCaMP6s signals evoked by electrical HDB stimulation, displayed as in **(A)**. HDB stimulation caused prolonged fluorescence increases in AON axon terminals (red trace). Black trace shows GCaMP6s signal from the same region of interest shortly after injection of muscimol into AON. AON inactivation with muscimol abolished inhalation- and HDB stimulation-evoked responses in the OB.

**Figure 4:**
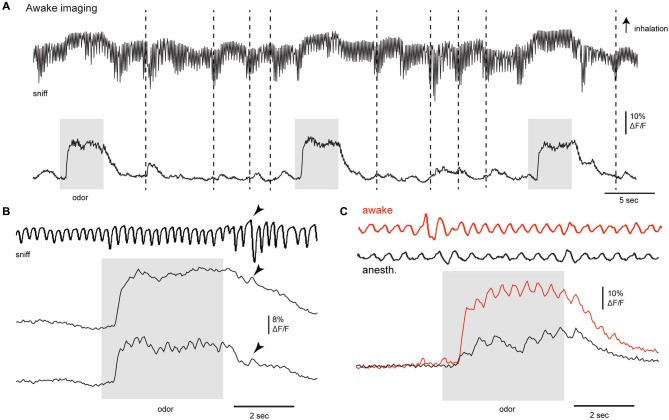
**Activation of centrifugal AON inputs to the olfactory bulb in the awake animal. (A–C)** GCaMP3 signals imaged from AON terminals in the dorsal OB of an awake animal after viral injection to the AON in Chrna7-Cre mice. **(A)** GCaMP3 signals imaged from the dorsal OB in an awake, head fixed mouse; odorant (0.5% of a mixture of 4 odorants: 2-hexanone, butyl acetate, ethyl butyrate, methyl valerate) is presented three times; activity from one ROI is displayed. Top trace in **(A, B and C)** shows respiration measured via thermocouple (placed in front of the nose in the depicted example), with inhalation up in all cases. Stippled lines indicate bouts of higher frequency sniffing; arrowhead points to a single deep inhalation. Odorant application as well as high-frequency sniffing in the absence of odorants activated AON inputs as reported with GCaMP3.

### Epifluorescence data analysis

Basic processing and analysis of optical signals followed protocols previously described for epifluorescence imaging from olfactory sensory neurons (OSNs; Wachowiak and Cohen, 2001; Verhagen et al., 2007; Wesson et al., 2008; Carey et al., 2009). Initial data processing included extracting fluorescence time-courses from visually-selected regions of interest (ROIs; ROIs consisted of 9–12 pixels and were distributed equally across activated areas on the dorsal OB) and upsampling of optical signals to 100 Hz to match the acquisition rate of respiratory signals. Repeated trials (3–8 trials) were averaged before analysis to improve signal to noise ratio.

For odorant response maps, ΔF/F values were calculated from temporal averages of 10 frames before odorant onset and 10 frames after the first inhalation of odorant. For display in the figures, maps were scaled from 0 to 95% of the maximal ΔF/F and pixel resolution doubled (to 512 × 512) using bilinear interpolation. Measurement of inhalation-evoked response amplitudes, peak odorant-evoked response amplitudes and signal onset latencies were made as described previously using a custom algorithm that fit the optical signals to a double sigmoid function (Wesson et al., 2008). The algorithm was modified slightly to fit the higher respiration frequencies in awake mice. Onset time was defined as the time the fitted optical signal reached 10% of its maximal amplitude (*t*_10_). Response onset latencies were measured relative to the start of the first inhalation after odorant onset. Latencies were measured from multiple foci and multiple odorants and averaged across inhalations of odorant before computing a grand average across animals. ROIs were counted as showing detectable inhalation-locked responses if the algorithm was able to fit responses to at least four sniffs in a given trial. All analyses were performed using custom software written in Matlab or LabVIEW. Summary data are reported as mean (or median) ± standard deviation (SD) unless noted otherwise. All statistical tests were performed using the Matlab statistics toolbox. An exponential function was used to compute τ_off_ values.

### Awake *in vivo* two photon imaging

Animals were prepared for two-photon imaging in the same manner as described for the awake head fixed epifluorescence imaging experiments, except that animal were habituated to run on a free floating Styrofoam ball. Imaging was carried out with a two-photon microscope (MOM; Sutter Instruments) coupled to a pulsed Ti:Sapphire laser (Mai Tai HP; Spectra-Physics) and controlled by Scanimage 3.9 (Pologruto et al., 2003). In all experiments, imaging was performed through a 16 × 0.8 numerical aperture objective (Nikon) and emitted light was collected by multialkali photomultiplier tubes (Hamamatsu R6357). Images were acquired at 3.7 Hz in most experiments. Fluorescence time series were extracted and analyzed with custom Matlab scripts, and ΔF/F was calculated as in *in vitro* experiments. Pseudocolor activation maps reflect an average of 8 trials in which movies were spatially filtered using a Gaussian window with a sigma of 0.75 pixels and temporally filtered using a fourth-order Butterworth filter with a cutoff frequency of 0.25 Hz. Correlation coefficients of odor evoked activity maps were calculated from pairwise correlations of averaged (8 odor presentations) ΔF maps.

### Inactivation of the AON

Microinjection of TTX (0.5 μl, 50 μM, Sigma-Aldrich) or muscimol (0.5 μl, 0.8 mM, Sigma-Aldrich) was performed using a glass pipette connected to a Picospritzer III (Parker Instruments). The tip of the glass pipette was lowered to the AON using the same stereotaxic coordinates as for viral vector injection. The glass pipette was left in the AON for approximately 5 min after the injection to allow for drug diffusion into the targeted structure. The amount of drug injected (approximately 10 ng and 50 ng for TTX and muscimol, respectively) is similar to that reported previously for *in vivo* inactivation (Zhuravin and Bures, 1991; Meyer and Louilot, 2012; Stratford and Wirtshafter, 2012).

### Histology

Transgene expression was evaluated with post hoc histology in all experiments to confirm accurate targeting of AON neurons and a lack of expression in OB neurons. Mice were deeply anesthetized with an overdose of sodium pentobarbital and perfused with phosphate-buffered saline (PBS) followed by 4% paraformaldehyde in PBS. Tissue was vibratome-sectioned as described previously (Wachowiak et al., 2013) and expression evaluated from native fluorescence without immunohistochemical amplification. For display, image stacks were obtained with an Olympus FV10i confocal laser scanning microscope.

## Results

### Expression of GCaMP in AON neurons

We used a mouse line, Chrna7-Cre, in which an IRES-Cre bi-cistronic cassette was introduced into the 3’noncoding region of the cholinergic nicotinic receptor alpha7 gene (Chrna7) (Rogers et al., 2012b) to achieve expression of genetically-encoded calcium reporters in neurons within the AON (Figure 1A). The nicotinic receptor alpha7 (α7) is expressed in both neuronal and nonneuronal tissues throughout the body and expression is especially high in AON neurons (Dominguez del Toro et al., 1994; Brunjes et al., 2005). Stereotaxic injection of the Cre-dependent viral vectors rAAV 2/1.FLEX.GCaMP3 (Atasoy et al., 2008; Tian et al., 2009; Betley and Sternson, 2011) or rAAV2/9.FLEX.GCaMP6s (Chen et al., 2013) centrally into the AON resulted in strong GCaMP expression in principal neurons in all major AON subdivisions (dorsal, lateral, medial and ventral part; expression in pars externa was not systematically analyzed, but observed in most animals; Figure 1B). The basic morphology of the labeled cells is dominated by one or more thick apical dendrites typical of pyramidal neurons (Figure 1C), in agreement with previous morphological descriptions of AON projection neurons (Brunjes and Kenerson, 2010).

GCaMP fluorescence was readily apparent in axonal projections from the AON to the OB, with expression predominately in the granule cell and external plexiform layers of the ipsilateral OB (Figure 1D) and fewer fibers in the external plexiform layer of the contralateral OB (Figure 1E), consistent with earlier characterizations of AON–OB projections (Reyher et al., 1988). Importantly, in the majority of animals AON infection resulted in no or only a few neuron somata in the OB expressing GCaMP (Figure 1D; the dense axonal terminations in the granule cell layer lead to the appearance of cellular labeling here due to a “shadowing” effect, but high-resolution confocal microscopy confirmed only sparse cellular expression). We were also able to drive expression of GCaMP in AON-OB projection neurons via retrograde infection with the same virus after injection into the OB of young (postnatal day 1–3) (7 mice) or adult Chrna7-Cre (2 mice) animals, as previously reported for cholecystokinin (CCK)-expressing neurons in AON (Rothermel et al., 2013). Retrograde infection via OB injection led to GCaMP expression in a large population of AON neurons on the ipsilateral side and fewer neurons in the contralateral AON, with expression in few if any neurons in the OB (not shown). As for the AON injections, sparsely labeled cells could be observed below the mitral cell layer as well as in the granule cell layer. Thus, projections from the AON to the OB appear to be largely comprised of α7—expressing neurons which can be selectively targeted with viral vectors, while infection of resident neurons in the postnatal OB appears to very sparse.

### Functional imaging of AON inputs to the OB *in vivo*

We next attempted to visualize the activation of AON projections to the dorsal OB as reported by GCaMP3 expression in their axon terminals. Using epifluorescence imaging focused slightly below the glomerular layer, electrical stimulation of the AON (50 pulses at 50 Hz, see Section Materials and Methods for details) evoked a large, transient increase in GCaMP3 fluorescence across the dorsal OB (Figure 2A). Electrically-evoked activity was evenly distributed over the dorsal surface of the OB in all recorded animals (Figure 2A). The mean stimulation-evoked increase across the dorsal OB was 21.6 ± 19.8% ΔF/F (*n* = 5 mice), with signals reaching as high as 80% ΔF/F in the strongest-activated areas. The minimum number of pulses necessary to evoke a response ranged between 4 and 8 pulses. AON stimulation-evoked GCaMP3 fluorescence increases decayed with a time-constant of 1.0 ± 0.16 s (*n* = 5 mice), comparable to that reported for the decay of GCaMP3 signals expressed in mitral and pyramidal cells after a brief spike burst (Wachowiak et al., 2013). The strong amplitude and rapid decay of this signal is consistent with it reflecting stimulation-evoked action potentials at the axon terminals of AON projections to the OB.

Next we asked whether sensory inputs to the OB are capable of activating feedback projections from AON. We and others have previously reported that inhalation alone can weakly activate olfactory sensory input to the OB and drive mitral/tufted cell activity (Grosmaitre et al., 2007; Carey et al., 2009; Wachowiak et al., 2013; Rothermel et al., 2014). In mice expressing GCaMP3 in AON projections (*n* = 8), we found that artificial inhalation of clean air at 1 Hz (see Section Materials and Methods) evoked small-amplitude fluorescence transients detectable above baseline noise in some regions (60 out of 144 ROIs, *n* = 301 sniffs, eight mice, see Section Materials and Methods for detection criteria). The mean peak amplitude of these detectable transients was 1.7 ± 0.9% ΔF/F. In five mice in which AON was also stimulated electrically, detectable inhalation-evoked signals reached a magnitude of 3.0 ± 2.2% of the magnitude of the AON stimulation response. This result suggests that even weak sensory inputs driven by inhalation are capable of driving descending AON inputs to the OB, and that these descending inputs are temporally patterned by inhalation.

Odorant presentation evoked larger GCaMP3 fluorescence changes that were, like the electrically-evoked signals, broadly but heterogeneously distributed across the dorsal OB (Figure 2B). The peak amplitude of odorant-evoked GCaMP3 signals reached up to 30% ΔF/F (mean across the imaged region, 5.6 ± 6.2%; *n* = 8 mice), and ranged from 1.9–36.5% of the peak response evoked by electrical AON stimulation (*n* = 5 mice). Responses to individual inhalations could be clearly resolved within the evoked GCaMP3 signals (Figure 2B). Odorant-evoked responses displayed a latency relative to inhalation onset of 234 ± 45 ms (*n* = 5 mice, 271 responses); these times were slower than previously-reported response latencies of OB interneurons (Wachowiak et al., 2013). To compare AON response latencies to those of primary sensory inputs imaged with the same optical reporter, we crossed OMP-Cre animals to the Ai38 GCaMP3 reporter line (Zariwala et al., 2012), which resulted in expression of GCaMP3 in olfactory sensory neuron axon terminals. In OMP-Cre:Ai38 mice, odorants evoked spatially organized, glomerular signals which showed robust inhalation-linked response dynamics (Figure 2C) as observed previously using synthetic calcium indicators and GCaMP2 (Wachowiak and Cohen, 2001; Bozza et al., 2004; Soucy et al., 2009; Ma et al., 2012; Wachowiak et al., 2013). Odorant-evoked response latencies relative to inhalation onset were 146 ± 30 ms (*n* = 2 mice, 32 responses), and so were substantially faster than those of AON projections to the OB (Figure 2D).

To ensure that odorant-evoked responses were not due to spurious GCaMP expression in neurons with the OB, we locally inactivated AON neurons by microinjecting tetrodotoxin (TTX). Local TTX application into the AON (50 μM, 0.5 μl) dramatically reduced odorant-evoked responses in the OB (Figure 3A, reduction to 13.2 ± 12.9% (median ± SD) of the peak ΔF under control conditions; *n* = 3 mice). Thus, sensory-evoked GCaMP signals in the OB originate largely, if not entirely, from AON feedback to the OB. Overall, these results suggest that AON input to the OB can be triggered by both weak and strong sensory input, is patterned by respiration, and may therefore provide rapid feedback to the OB that is updated with each inhalation.

### Basal forebrain activation also drives input from AON to the OB

In addition to ascending sensory signals from the OB, the AON receives centrifugal input from higher-order brain areas including classical neuromodulatory centers. The horizontal limb of the HDB, for example, sends cholinergic and GABA-ergic projections to the OB, piriform cortex and AON (Woolf et al., 1984; Linster et al., 1999; Zaborszky et al., 2012; Rothermel et al., 2014). To assess whether neuromodulatory centers might modulate OB activity via their impact on AON projections, we investigated the effects of HDB stimulation on AON inputs to the OB. Brief electrical stimulation of HDB (same stimulus parameters as for AON stimulation; see Section Materials and Methods) evoked a modest increase in GCaMP3 fluorescence that outlasted the stimulus train by as much as 10 s (*n* = 4 mice; Figure 3B). The peak amplitude of HDB stimulation-evoked GCaMP3 signals reached up to 12% ΔF/F (mean, 3.1 ± 2.7%; *n* = 4 mice), corresponding to 4.9–9.8% of the maximal AON stimulation-evoked response observed in the same animals (*n* = 2 mice). The prolonged effect of HDB stimulation-evoked GCaMP3 fluorescence increases suggests a modulation of ongoing AON output rather than a direct excitation of AON projection neurons or direct cholinergic activation of AON axon terminals in the OB. To confirm this, in one animal we locally inactivated AON neurons by microinjecting the GABA_A_ receptor agonist muscimol into AON. We predicted that muscimol should inactivate AON throughput without affecting axons of passage projecting directly from HDB to the OB. As expected, muscimol injection into the AON (0.8 mM, 0.5 μl) eliminated HDB stimulation-evoked responses in the OB (Figure 3B). Muscimol also eliminated inhalation- and odor-evoked signals in the OB. Together these results indicate that basal forebrain, in addition to the known direct projections from basal forebrain to the OB (Macrides et al., 1981; Shipley and Adamek, 1984; Rothermel et al., 2013, 2014), can indirectly modulate OB processing via its enhancement of AON inputs to the OB.

### Sensory-evoked AON feedback to the OB imaged in the awake mouse

Centrifugal inputs from higher-order centers can play a major role in shaping processing in primary sensory areas as a function of behavioral state. We thus imaged AON inputs to the OB during passive and active sampling of olfactory stimuli in the awake, head-fixed mouse using epifluorescence and two-photon imaging. Under epifluorescence, odorants robustly activated AON projections to the OB during wakefulness, with peak GCaMP3 signals reaching up to 18% ΔF/F (*n* = 2 mice). Odorant-evoked responses were reliable across repeated odorant presentations and modulated by respiration (Figures 4A,B). While the degree of respiratory modulation appeared smaller than in the anesthetized mouse, transient GCaMP3 signals were nonetheless clearly linked to inhalation (Figures 4B,C). Bouts of higher frequency sniffing (Figure 4A, stippled lines) or even a single strong inhalation (arrowheads) transiently activated AON inputs to the OB even in the absence of odorant, consistent with the results of the artificial inhalation experiments.

We also found evidence that the strength of AON input to the OB was itself modulated by wakefulness. In order to compare responses in the awake versus anesthetized states, we briefly (1–3 min) anesthetized head-fixed mice with isoflurane, then imaged GCaMP signals as the animal returned to the waking state over the next 1–2 min (Wachowiak et al., 2013). Odorant-evoked GCaMP signals under anesthesia were significantly smaller than those observed during wakefulness (Figure 4C), with peak amplitudes just after removal of isoflurane being reduced to 34 and 25% of those before anesthesia (*n* = 2 mice). This result is in contrast to what we and others have observed for OB output neurons, where mitral/tufted cell responses imaged with GCaMPs are larger under anesthesia than during wakefulness. These results suggest that the strength of sensory-evoked descending inputs from AON to the OB may itself vary with brain state.

**Figure 5:**
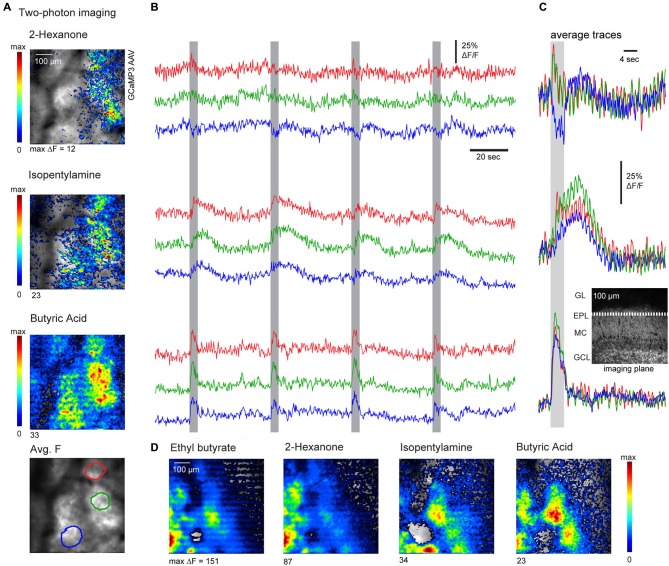
**Two-photon imaging reveals odorant-specific patterns of AON feedback to the OB. (A)** Resting fluorescence (grayscale) and pseudocolor overlay of odorant-evoked GCaMP3 fluorescence changes imaged with two-photon microscopy in an awake, head-fixed mouse. Odorant-evoked responses were broadly but heterogeneously distributed and varied across odorants. Bottom: Regions of interest (ROIs) used for the time-courses shown in **(B)**. **(B)** Traces showing the time-course of GCaMP3 signals imaged from different ROIs during four consecutive odorant presentations. Odorant presentation elicited reproducible responses which differed in different ROIs. Responses also differed in their temporal dynamics and could also include fluorescence decreases. **(C)** Average odorant-evoked signals from the same ROIs and same odorants as in **(A)** and **(B)**, averaged over eight trials. Inset: approximate imaging plane shown in a reference section. **(D)** Odorant-evoked response maps imaged with two-photon microscopy in a different animal, again showing odorant-specific patterns of fluorescence signals (GCaMP3).

Next we further evaluated the spatiotemporal organization of AON feedback to the OB using two-photon imaging in the awake mouse (see Section Materials and Methods for details). Imaging was performed at a single focal plane set at the superficial external plexiform layer (see insert, Figure 5C), which receives dense axonal projections from AON (also compare to Figure 1D). Apparent foci in the average projection image at the bottom of Figure 5A are artifacts from anatomical features (blood vessels). We imaged responses to several odorants *per se*ssion, presenting each odorant eight times with an inter-stimulus interval of 36 s. Figure 5 shows response maps evoked by three sample odorants (Figure 5A) as well as example traces depicting the fluorescence signals recorded from three ROIs (Figure 5B), using these odorants. Odorant-evoked responses were reliably repeated across multiple presentations and showed peak amplitudes of up to 117% ΔF/F (mean peak response across all odorants, 35.8 ± 27.2%; *n* = 3 mice, 52 odorant responses). Notably, odorant-evoked responses had diverse temporal characteristics that were reproducible for individual odorants but substantially differed between them (Figure 5C; compare the prolonged odor response to isopentamylamine to the brief response evoked by butyric acid). The same odorant could also evoke distinct temporal response patterns in different areas of the imaged region (for example, compare red and green trace to blue trace for 2-hexanone). Response maps of AON input activity also revealed that different odorants could evoke distinct spatial patterns of feedback activity (Figures 5A,D). To quantify this spatial heterogeneity, we correlated averaged response maps evoked by different odorants or by blocks of repeated trials of the same odorant. The correlation coefficients between responses to different odorants (*r* = 0.54 ± 0.17, 69 pair-wise comparisons, 11 imaging sessions, *n* = 3 mice) were significantly lower than correlation coefficients calculated for trials of the same odorant (*r* = 0.73 ± 0.14, 5 pair-wise comparisons, 3 imaging sessions, *p* = 0.03 Mann–Whitney *U*-test), indicating some degree of spatial specificity in AON feedback to the OB. Overall, these results suggest that sensory-evoked AON feedback to the OB is not homogenous, but instead displays distinct spatiotemporal patterns evoked by different odorants.

### AON mediates odorant-specific feedback to the contralateral OB

**Figure 6:**
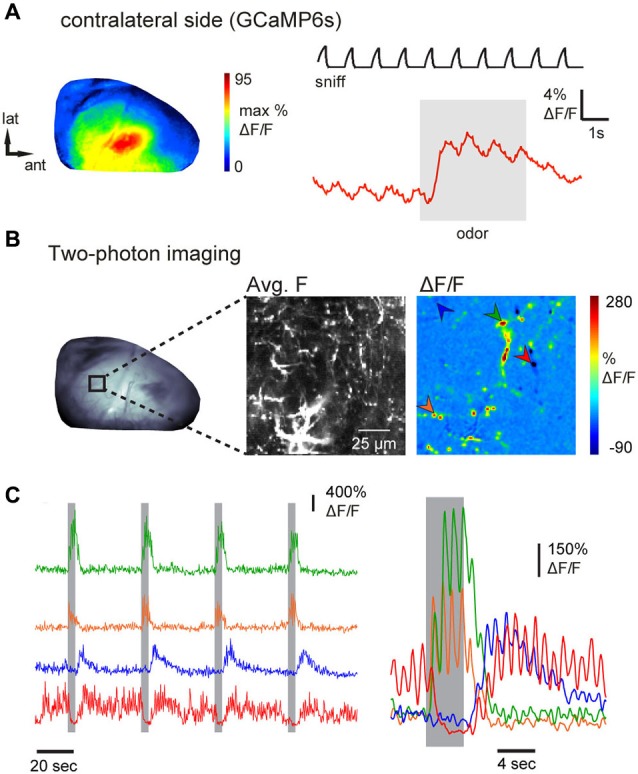
**Inhalation-driven and odorant-specific centrifugal AON inputs to the contralateral OB. (A)** Response map (left) and time-course (right) of odorant-evoked (1% ethyl butyrate) GCaMP6s signals imaged from AON terminals in the contralateral dorsal OB of an anesthetized mouse, displayed as in Figure 2. As with ipsilateral signals, contralateral AON input responses were driven by inhalation and broadly distributed across the dorsal OB. **(B)** Contralateral AON inputs imaged with two-photon microscopy in an anesthetized mouse. Left: epifluorescence image of the dorsal OB. Middle: Two-photon images showing resting GCaMP6s fluorescence (average fluorescence map) in AON axons in the indicated region. Right: Odorant-evoked response map showing strong fluorescence changes in small, sparsely-distributed foci likely corresponding to individual axonal fibers and varicosities. Arrowheads indicate foci used for the time-courses shown in **(C)**. **(C)** Traces demonstrating diverse responses of different contralateral AON inputs. Left traces show continuous fluorescence signals taken from the regions indicated in **(B)** during four consecutive odorant presentations. Right traces show the same responses averaged across eight trials. Fluctuations indicate inhalation-driven fluorescence increases (sniff trace not shown). Responses were consistent across trials and included “off” responses (blue trace) as well as suppression of ongoing activity (red trace).

The AON has been proposed to mediate the communication of olfactory information between each OB via its projections to the contralateral OB (Schoenfeld and Macrides, 1984; Lei et al., 2006; Yan et al., 2008; Kikuta et al., 2010; Kay and Brunjes, 2014). Thus, in a final experiment, we attempted to image from individual axons projecting from AON to the contralateral OB. Epifluorescence imaging in an anesthetized mouse revealed broadly distributed and inhalation-driven odorant evoked GCaMP6s fluorescence signals that were qualitatively similar to those observed in ipsilateral imaging (Figure 6A). Because contralateral projections showed sparser labeling than ipsilateral projections in all preparations (e.g., Figure 1E), discrete axons and axonal varicosities could be visualized using two-photon imaging in the deep external plexiform layer of the contralateral OB (Figure 6B, average fluorescence map), allowing us to map odorant-evoked feedback from individual contralaterally-projecting axons (Figure 6B). Examples of GCaMP6s signals recorded from four ROI centered on putative axon terminals are shown in Figure 6C. Odorants evoked responses in distinct combinations of axon terminals with temporally diverse responses that included “simple” fluorescence increases during odorant stimulation, “off” responses, as well as apparent suppression of ongoing activity (Figure 6C). In each case, responses were repeated across successive odorant presentations. These results indicate that AON sends odorant-specific feedback signals to both ipsi- and contralateral OBs.

## Discussion

Olfactory cortex sends strong feedback projections to the OB, the first stage of synaptic processing in the olfactory system. While this cortical feedback has been shown to profoundly modulate OB output and hypothesized to play an integral role in olfactory processing in the awake animal (Strowbridge, 2009; Boyd et al., 2012; Markopoulos et al., 2012; Soria-Gómez et al., 2014), the functional properties of feedback to the OB from any neuronal population have yet to be characterized *in vivo*. Centrifugal projections from the AON—a simplified cortical structure interconnected with the OB and piriform cortex—are among the most numerous of centrifugal inputs to the OB (Carson, 1984; Shipley and Adamek, 1984). Here, we selectively labeled descending AON projection neurons and imaged activation of their axon terminals in the OB, an approach similar to recent studies imaging from axonal processes in visual or somatosensory cortex (Petreanu et al., 2012; Glickfeld et al., 2013). We found that Cre-dependent viral vector injection into Chrna7-Cre animals was sufficient to drive GCaMP expression in principal neurons throughout the AON. GCaMP expression in AON was sufficient to report the arrival of action potentials at their axon terminals in the OB. AON neurons could be labeled either by direct virus injection into the AON or by retrograde viral transport after virus injection into the OB, consistent with our recent report of the retrograde infection capabilities of many recombinant AAV vectors (Rothermel et al., 2013). In either case, a key feature of our approach was the ability to selectively image the activation of centrifugal projections to the OB. The AON is composed of at least five major subdivisions which appear functionally distinct on the basis of their anatomical projections, intrinsic cellular makeup and chemoarchitecture (Reyher et al., 1988; Brunjes et al., 2005; Meyer et al., 2006; Illig and Eudy, 2009; Kay and Brunjes, 2014). While clearly a simplification, as a first step we did not attempt to selectively investigate these subdivisions and instead considered AON output signals as a whole.

There are several lines of evidence indicating that the fluorescence signals imaged from the dorsal OB in our experiments originated largely or entirely from GCaMP expressed in centrifugal projections from the AON. First, even with direct bulbar virus injections we observed only a very sparse cellular labeling in the OB which was consistent with the sparse distribution of Chrna7-expressing neurons in adult mice using transgenic markers (The Gene Expression Nervous System Atlas (GENSAT) Project^1^) (Gong et al., 2003); virus injection into AON, as used in the majority of experiments, should further minimize the chances of expression in OB neurons. Second, strong and short-latency responses could be triggered by direct electrical stimulation of the AON. Third, odorant-evoked responses imaged from the OB showed longer response latencies relative to inhalation compared to responses imaged from olfactory sensory neuron axon terminals expressing the same GCaMP3 reporter (i.e., Figure 2D), and also slightly longer than those reported for OB interneurons and mitral/tufted cells (Wachowiak et al., 2013), as would be expected for a sensory-evoked feedback projection. Finally, TTX and muscimol blockade experiments confirmed the AON as the major signal source for fluorescence activity measured at the dorsal OB.

While AON projections to the OB have been well-described anatomically (Brunjes et al., 2005), their functional properties *in vivo* have, until now, been completely uncharacterized. For example, earlier studies have proposed that AON pars externa mediates rapid feedback of OB output to the contralateral OB (Schoenfeld and Macrides, 1984; Yan et al., 2008), a prediction now confirmed by our results. In addition, our experiments provide the first functional evidence in support of an indirect modulation of OB function mediated by higher-order areas such as basal forebrain, as predicted from earlier anatomical findings (Zaborszky et al., 2012; Rothermel et al., 2014). Other results—for example, the strong respiratory coupling of AON feedback neurons to the ipsilateral OB and the rich diversity of odorant response specificities and response polarities apparent with high-resolution two-photon imaging—are not easily predicted from anatomical studies. The implications of such findings for the functional role of the AON in olfactory processing are discussed in more detail below.

### Sensory-evoked feedback projections from AON to the OB

Odorants evoked fluorescence changes that were transient and driven by inhalation, suggesting that even in the anesthetized animal there are strong sensory-evoked feedback projections from AON to the OB. Rapid inhalation-driven bursts of AON input to the OB were also robust in the awake mouse. The persistence of strong respiratory coupling in AON feedback to the OB is somewhat surprising given that OB output neurons show diverse temporal responses which span the respiratory cycle (Chaput, 1986; Carey and Wachowiak, 2011; Shusterman et al., 2011). One explanation for this result may be that the AON receives input preferentially from OB tufted (as opposed to mitral) cells (Haberly and Price, 1977; Scott et al., 1980; Scott, 1981; Macrides and Schneider, 1982; Nagayama et al., 2010; Sosulski et al., 2011). Functionally, tufted cells are more excitable, have higher firing frequencies and display stronger respiratory locking compared to mitral cells (Schneider and Scott, 1983; Ezeh et al., 1993; Nagayama et al., 2004; Griff et al., 2008; Burton and Urban, 2014) and so may preserve the timing of odor sampling more faithfully across their population. Inhalation-coupled feedback from AON may play several roles in shaping how the OB processes incoming olfactory information. First, inhalation-coupled feedback may provide a real-time report of sensory input referenced to the respiratory cycle. Inhalation alone evokes weak OSN inputs to the OB (Grosmaitre et al., 2007; Carey et al., 2009), which are presumably relayed to the AON and which may, as our recordings suggest, trigger inhalation-coupled AON feedback even in the absence of odorant. Since AON projection terminals innervate all major OB layers, inhalation-coupled AON activity may impose a feedback signal on OB circuits that can serve as a reference for the timing of the respiratory cycle across different OB cells types; this timing signal may be important in shaping the odor-specific, respiratory patterning of mitral/tufted cell dynamics that is thought to be important in encoding odor information (Bathellier et al., 2008; Cury and Uchida, 2010; Shusterman et al., 2011).

The circuit mechanisms underlying rapid, sensory-evoked feedback to the OB remain to be elucidated. So far, only one study has selectively investigated the influence of centrifugal AON inputs on defined OB circuits including OB output neurons (Markopoulos et al., 2012). Using optical AON stimulation, Markopoulos et al. (2012) found evidence that AON inputs drive a fast direct depolarization of mitral/tufted cells as well as a delayed disynaptic inhibition mainly mediated by granule cells. AON effects on mitral/tufted cells were independent of the exact phase of the respiration but strongly dependent on mitral/tufted cell basal firing rate. The authors concluded that AON feedback might shape OB output by creating a window of opportunity for mitral/tufted cell spikes and enforcing a broad inhibition that could suppress background activity. Our observation that AON feedback activity shows strong modulations with the respiration cycle is consistent with this hypothesis and supports the idea that the timing of cortical feedback projections to the OB is important for modulating OB activity.

High-resolution two-photon imaging revealed that, on a small spatial scale, AON feedback to the OB shows considerable diversity in odorant specificity and temporal dynamics, with different individual axons—or small populations of axons—showing distinct odorant response profiles and even distinct combinations of excitatory and suppressive responses. These results suggest an unforeseen richness in the representation of odor information carried by centrifugal feedback to the OB. The logic of such specific feedback projections remains to be explored; however these results suggest that AON feedback functions as more than just a global, activity-dependent regulator of OB output. One possibility is that AON feedback might selectively modulate sensory inputs to different functional domains of the OB. It has been reported that the AON is (in part) topographically organized with AON outputs to the OB showing a dorsal to ventral topography and different AON subdivisions targeting different OB layers (Davis and Macrides, 1981; Luskin and Price, 1983; Reyher et al., 1988; Brunjes et al., 2005). Ascending projections from the OB to the AON (both pars externa and pars principalis) also maintain a rough topographic organization (Schoenfeld et al., 1985; Scott et al., 1985; Ghosh et al., 2011; Miyamichi et al., 2011), and different odorants activate topographically distinct groups of neuron in pars externa (but not pars pricipalis) (Kay et al., 2011). It is thus possible that centrifugal projections from the AON may modulate neural activity in homotopic OB areas, meaning that domains within the OB that were activated by a specific odorant could receive selective and targeted feedback from the AON. Sensory-evoked feedback from the AON that targets specific OB regions would be in a powerful position to selectively modulate the gain of particular functional domains and therefore dynamically adjust the relative responsiveness of OB output to different odorant classes. Odorant-specific feedback to the OB might also play a role in experience-dependent plasticity of early odor representations—for example, mediating glomerulus-specific changes in response gain for glomeruli involved in representing odor objects associated with aversive or appetitive stimuli (Kass et al., 2013; Abraham et al., 2014). While speculative at this point, such a role would be consistent with that proposed earlier for AON on the basis of its anatomical connections with the OB and piriform cortex (Haberly, 2001).

### The AON as a mulifunctional hub for shaping early olfactory processing

In addition to being activated by incoming olfactory information, we also found that AON projections to the OB were activated by stimulating basal forebrain, illustrating that the AON functions not only as a low-level feedback center or a relay nucleus but also as a mediator of “top-down” signals from higher-order areas. This finding is consistent with anatomical studies showing that the AON not only receives direct input from the OB but also from diverse brain areas including basal forebrain (Zaborszky et al., 2012; Rothermel et al., 2014), piriform cortex (Haberly and Price, 1978; Luskin and Price, 1983; Hagiwara et al., 2012) and the contralateral AON (Brunjes et al., 2005). Thus, the role of the AON in modulating incoming olfactory information may be analogous to pre-cortical nuclei in other sensory systems. In the auditory system for example, olivocochlear efferents mediate central control of the sensitivity of hair cells to external sounds (reviewed in Rabbitt and Brownell, 2011). This system can be activated by auditory input from the cochlear nucleus, thus constituting a rapid sensory feedback pathway, but can also be driven by “top-down” signals arising, for example, from the vocal motor system to modulate auditory sensitivity to self-generated sounds (Weeg et al., 2005).

We hypothesize that the AON serves a similar multifunctional role, acting as modulatory hub that integrates incoming sensory information with inputs from other brain areas in order to rapidly shape OB output according to ongoing olfactory sampling as well as overall behavioral state. The AON likely also plays an important role in olfactory processing by shaping odor representations as they ascend to other cortical areas; indeed, there are robust projections from the AON to anterior piriform cortex (Hagiwara et al., 2012), and AON neurons display unique odorant response properties consistent with their integrating and transforming sensory inputs from the OB (Lei et al., 2006; Kikuta et al., 2010; Kay et al., 2011). Whether these multiple functional pathways are mediated by separate or overlapping circuits, or mediated by distinct subdivisions of AON, remains unexplored. Genetically-targeted, optical approaches similar to those used here to functionally characterize, for the first time, centrifugal AON projections to the OB should be useful in further dissecting these pathways in order to understand how each is engaged to shape olfactory processing during behavior.

## Author contributions

Markus Rothermel and Matt Wachowiak designed the experiments, Markus Rothermel performed the imaging experiments and data analysis, Markus Rothermel and Matt Wachowiak wrote the paper.

## Conflict of interest statement

The authors declare that the research was conducted in the absence of any commercial or financial relationships that could be construed as a potential conflict of interest.

## References

[B1] Abraham N. M. Vincis R. Lagier S. Rodriguez I. Carleton A. Eichenbaum H. (2014). Long term functional plasticity of sensory inputs mediated by olfactory learning. Elife 3:e02109 10.7554/elife.02109 24642413 PMC3953949

[B2] Atasoy D. Aponte Y. Su H. H. Sternson S. M. (2008). A FLEX switch targets Channelrhodopsin-2 to multiple cell types for imaging and long-range circuit mapping. J. Neurosci. 28, 7025–7030 10.1523/JNEUROSCI.1954-08.2008 18614669 PMC2593125

[B3] Bathellier B. Buhl D. L. Accolla R. Carleton A. (2008). Dynamic ensemble odor coding in the mammalian olfactory bulb: sensory information at different timescales. Neuron 57, 586–598 10.1016/j.neuron.2008.02.011 18304487

[B4] Betley J. N. Sternson S. M. (2011). Adeno-associated viral vectors for mapping, monitoring and manipulating neural circuits. Hum. Gene Ther. 22, 669–677 10.1089/hum.2010.204 21319997 PMC3107581

[B5] Boyd A. M. Sturgill J. F. Poo C. Isaacson J. S. (2012). Cortical feedback control of olfactory bulb circuits. Neuron 76, 1161–1174 10.1016/j.neuron.2012.10.020 23259951 PMC3725136

[B6] Bozza T. Mcgann J. P. Mombaerts P. Wachowiak M. (2004). In vivo imaging of neuronal activity by targeted expression of a genetically encoded probe in the mouse. Neuron 42, 9–21 10.1016/S0896-6273(04)00144-8 15066261

[B7] Broadwell R. D. Jacobowitz D. M. (1976). Olfactory relationships of the telencephalon and diencephalon in the rabbit. III. The ipsilateral centrifugal fibers to the olfactory bulbar and retrobulbar formations. J. Comp. Neurol. 170, 321–345 10.1002/cne.901700305 62770

[B8] Brunjes P. C. Illig K. R. Meyer E. A. (2005). A field guide to the anterior olfactory nucleus (cortex). Brain Res. Brain Res. Rev. 50, 305–335 10.1016/j.brainresrev.2005.08.005 16229895

[B9] Brunjes P. C. Kenerson M. C. (2010). The anterior olfactory nucleus: quantitative study of dendritic morphology. J. Comp. Neurol. 518, 1603–1616 10.1002/cne.22293 20187150 PMC3546507

[B10] Burton S. D. Urban N. N. (2014). Greater excitability and firing irregularity of tufted cells underlies distinct afferent-evoked activity of olfactory bulb mitral and tufted cells. J. Physiol. 592(Pt. 10), 2097–2118 10.1113/jphysiol.2013.269886 24614745 PMC4227897

[B11] Canteras N. S. Simerly R. B. Swanson L. W. (1995). Organization of projections from the medial nucleus of the amygdala: a PHAL study in the rat. J. Comp. Neurol. 360, 213–245 10.1002/cne.903600203 8522644

[B12] Carey R. M. Verhagen J. V. Wesson D. W. Pirez N. Wachowiak M. (2009). Temporal structure of receptor neuron input to the olfactory bulb imaged in behaving rats. J. Neurophysiol. 101, 1073–1088 10.1152/jn.90902.2008 19091924 PMC2657066

[B13] Carey R. M. Wachowiak M. (2011). Effect of sniffing on the temporal structure of mitral/tufted cell output from the olfactory bulb. J. Neurosci. 31, 10615–10626 10.1523/JNEUROSCI.1805-11.2011 21775605 PMC3159407

[B14] Carnes K. M. Fuller T. A. Price J. L. (1990). Sources of presumptive glutamatergic/aspartatergic afferents to the magnocellular basal forebrain in the rat. J. Comp. Neurol. 302, 824–852 10.1002/cne.903020413 1982006

[B15] Carson K. A. (1984). Quantitative localization of neurons projecting to the mouse main olfactory bulb. Brain Res. Bull. 12, 629–634 10.1016/0361-9230(84)90143-6 6206926

[B16] Chaput M. A. (1986). Respiratory-phase-related coding of olfactory information in the olfactory bulb of awake freely-breathing rabbits. Physiol. Behav. 36, 319–324 10.1016/0031-9384(86)90023-5 3961008

[B17] Chen T.-W. Wardill T. J. Sun Y. Pulver S. R. Renninger S. L. Baohan A. (2013). Ultrasensitive fluorescent proteins for imaging neuronal activity. Nature 499, 295–300 10.1038/nature12354 23868258 PMC3777791

[B18] Cury K. M. Uchida N. (2010). Robust odor coding via inhalation-coupled transient activity in the mammalian olfactory bulb. Neuron 68, 570–585 10.1016/j.neuron.2010.09.040 21040855

[B19] Davis B. J. Macrides F. (1981). The organization of centrifugal projections from the anterior olfactory nucleus, ventral hippocampal rudiment and piriform cortex to the main olfactory bulb in the hamster: an autoradiographic study. J. Comp. Neurol. 203, 475–493 10.1002/cne.902030310 6274922

[B20] Davis B. J. Macrides F. Youngs W. M. Schneider S. P. Rosene D. L. (1978). Efferents and centrifugal afferents of the main and accessory olfactory bulbs in the hamster. Brain Res. Bull. 3, 59–72 10.1016/0361-9230(78)90062-x 75756

[B21] De Carlos J. A. Lopez-Mascaraque L. Valverde F. (1989). Connections of the olfactory bulb and nucleus olfactorius anterior in the hedgehog (Erinaceus europaeus): fluorescent tracers and HRP study. J. Comp. Neurol. 279, 601–618 10.1002/cne.902790408 2918089

[B22] de Olmos J. Hardy H. Heimer L. (1978). The afferent connections of the main and the accessory olfactory bulb formations in the rat: an experimental HRP-study. J. Comp. Neurol. 181, 213–244 10.1002/cne.901810202 690266

[B23] Dominguez del Toro E. Juiz J. M. Peng X. Lindstrom J. Criado M. (1994). Immunocytochemical localization of the alpha 7 subunit of the nicotinic acetylcholine receptor in the rat central nervous system. J. Comp. Neurol. 349, 325–342 10.1002/cne.903490302 7852628

[B24] Doucette W. Restrepo D. (2008). Profound context-dependent plasticity of mitral cell responses in olfactory bulb. PLoS Biol. 6:e258 10.1371/journal.pbio.0060258 18959481 PMC2573932

[B25] Ezeh P. I. Wellis D. P. Scott J. W. (1993). Organization of inhibition in the rat olfactory bulb external plexiform layer. J. Neurophysiol. 70, 263–274 8395579 10.1152/jn.1993.70.1.263

[B26] Fu Y. Tucciarone J. M. Espinosa J. S. Sheng N. Darcy D. P. Nicoll R. A. (2014). A cortical circuit for gain control by behavioral state. Cell 156, 1139–1152 10.1016/j.cell.2014.01.050 24630718 PMC4041382

[B27] Gahring L. C. Enioutina E. Y. Myers E. J. Spangrude G. J. Efimova O. V. Kelley T. W. (2013). Nicotinic receptor alpha7 expression identifies a novel hematopoietic progenitor lineage. PLoS One 8:e57481 10.1371/journal.pone.0057481 23469197 PMC3586088

[B28] Gaykema R. P. Luiten P. G. Nyakas C. Traber J. (1990). Cortical projection patterns of the medial septum-diagonal band complex. J. Comp. Neurol. 293, 103–124 10.1002/cne.902930109 2312788

[B29] Ghosh S. Larson S. D. Hefzi H. Marnoy Z. Cutforth T. Dokka K. (2011). Sensory maps in the olfactory cortex defined by long-range viral tracing of single neurons. Nature 472, 217–220 10.1038/nature09945 21451523

[B30] Glickfeld L. L. Andermann M. L. Bonin V. Reid R. C. (2013). Cortico-cortical projections in mouse visual cortex are functionally target specific. Nat. Neurosci. 16, 219–226 10.1038/nn.3300 23292681 PMC3808876

[B31] Gomez D. M. Newman S. W. (1992). Differential projections of the anterior and posterior regions of the medial amygdaloid nucleus in the Syrian hamster. J. Comp. Neurol. 317, 195–218 10.1002/cne.903170208 1573064

[B32] Gong S. Zheng C. Doughty M. L. Losos K. Didkovsky N. Schambra U. B. (2003). A gene expression atlas of the central nervous system based on bacterial artificial chromosomes. Nature 425, 917–925 10.1038/nature02033 14586460

[B33] Griff E. R. Mafhouz M. Chaput M. A. (2008). Comparison of identified mitral and tufted cells in freely breathing rats: II. Odor-evoked responses. Chem. Senses 33, 793–802 10.1093/chemse/bjn040 18640966

[B34] Grosmaitre X. Santarelli L. C. Tan J. Luo M. Ma M. (2007). Dual functions of mammalian olfactory sensory neurons as odor detectors and mechanical sensors. Nat. Neurosci. 10, 348–354 10.1038/nn1856 17310245 PMC2227320

[B35] Haberly L. B. (2001). Parallel-distributed processing in olfactory cortex: new insights from morphological and physiological analysis of neuronal circuitry. Chem. Senses 26, 551–576 10.1093/chemse/26.5.551 11418502

[B36] Haberly L. B. Price J. L. (1977). The axonal projection patterns of the mitral and tufted cells of the olfactory bulb in the rat. Brain Res. 129, 152–157 10.1016/0006-8993(77)90978-7 68803

[B37] Haberly L. B. Price J. L. (1978). Association and commissural fiber systems of the olfactory cortex of the rat. J. Comp. Neurol. 178, 711–740 10.1002/cne.901780408 632378

[B38] Hagiwara A. Pal S. K. Sato T. F. Wienisch M. Murthy V. N. (2012). Optophysiological analysis of associational circuits in the olfactory cortex. Front. Neural Circuits 6:18 10.3389/fncir.2012.00018 22529781 PMC3329886

[B39] Ichikawa T. Hirata Y. (1986). Organization of choline acetyltransferase-containing structures in the forebrain of the rat. J. Neurosci. 6, 281–292 3944622 10.1523/JNEUROSCI.06-01-00281.1986 PMC6568606

[B40] Illig K. R. Eudy J. D. (2009). Contralateral projections of the rat anterior olfactory nucleus. J. Comp. Neurol. 512, 115–123 10.1002/cne.21900 18973225 PMC2587515

[B41] Karpov A. P. (1980). “Analysis of neuron activity in the rabbit’s olfactory bulb during food-acquisition behavior,” in Neural Mechanisms of Goal-Directed Behavior and Learning, eds Thompson R. F. Hicks L. H. Shvyrkov V. B. (New York: Academic Press), 273–282

[B42] Kass M. D. Rosenthal M. C. Pottackal J. Mcgann J. P. (2013). Fear learning enhances neural responses to threat-predictive sensory stimuli. Science 342, 1389–1392 10.1126/science.1244916 24337299 PMC4011636

[B43] Kato H. K. Chu M. W. Isaacson J. S. Komiyama T. (2012). Dynamic sensory representations in the olfactory bulb: modulation by wakefulness and experience. Neuron 76, 962–975 10.1016/j.neuron.2012.09.037 23217744 PMC3523713

[B44] Kay R. B. Brunjes P. C. (2014). Diversity among principal and GABAergic neurons of the anterior olfactory nucleus. Front. Cell. Neurosci. 8:111 10.3389/fncel.2014.00111 24808826 PMC4010738

[B45] Kay L. M. Laurent G. (1999). Odor- and context-dependent modulation of mitral cell activity in behaving rats. Nat. Neurosci. 2, 1003–1009 10.1038/14801 10526340

[B46] Kay R. B. Meyer E. A. Illig K. R. Brunjes P. C. (2011). Spatial distribution of neural activity in the anterior olfactory nucleus evoked by odor and electrical stimulation. J. Comp. Neurol. 519, 277–289 10.1002/cne.22519 21165975 PMC3342756

[B47] Kikuta S. Sato K. Kashiwadani H. Tsunoda K. Yamasoba T. Mori K. (2010). Neurons in the anterior olfactory nucleus pars externa detect right or left localization of odor sources. Proc. Natl. Acad. Sci. U S A 107, 12363–12368 10.1073/pnas.1003999107 20616091 PMC2901466

[B48] Lei H. Mooney R. Katz L. C. (2006). Synaptic integration of olfactory information in mouse anterior olfactory nucleus. J. Neurosci. 26, 12023–12032 10.1523/jneurosci.2598-06.2006 17108176 PMC6674854

[B49] Li J. Ishii T. Feinstein P. Mombaerts P. (2004). Odorant receptor gene choice is reset by nuclear transfer from mouse olfactory sensory neurons. Nature 428, 393–399 10.1038/nature02433 15042081

[B50] Linster C. Wyble B. P. Hasselmo M. E. (1999). Electrical stimulation of the horizontal limb of the diagonal band of broca modulates population EPSPs in piriform cortex. J. Neurophysiol. 81, 2737–2742 10368393 10.1152/jn.1999.81.6.2737

[B51] Luiten P. G. Gaykema R. P. Traber J. Spencer D. G. Jr. (1987). Cortical projection patterns of magnocellular basal nucleus subdivisions as revealed by anterogradely transported Phaseolus vulgaris leucoagglutinin. Brain Res. 413, 229–250 10.1016/0006-8993(87)91014-6 3300852

[B52] Luskin M. B. Price J. L. (1983). The topographic organization of associational fibers of the olfactory system in the rat, including centrifugal fibers to the olfactory bulb. J. Comp. Neurol. 216, 264–291 10.1002/cne.902160305 6306065

[B53] Ma L. Qiu Q. Gradwohl S. Scott A. Yu E. Q. Alexander R. (2012). Distributed representation of chemical features and tunotopic organization of glomeruli in the mouse olfactory bulb. Proc. Natl. Acad. Sci. U S A 109, 5481–5486 10.1073/pnas.1117491109 22431605 PMC3325716

[B54] Macrides F. Davis B. J. Youngs W. M. Nadi N. S. Margolis F. L. (1981). Cholinergic and catecholaminergic afferents to the olfactory bulb in the hamster: a neuroanatomical, biochemical and histochemical investigation. J. Comp. Neurol. 203, 495–514 10.1002/cne.902030311 6274923

[B55] Macrides F. Schneider S. P. (1982). Laminar organization of mitral and tufted cells in the main olfactory bulb of the adult hamster. J. Comp. Neurol. 208, 419–430 10.1002/cne.902080410 7119169

[B56] Markopoulos F. Rokni D. Gire D. H. Murthy V. N. (2012). Functional properties of cortical feedback projections to the olfactory bulb. Neuron 76, 1175–1188 10.1016/j.neuron.2012.10.028 23259952 PMC3530161

[B57] Matsutani S. (2010). Trajectory and terminal distribution of single centrifugal axons from olfactory cortical areas in the rat olfactory bulb. Neuroscience 169, 436–448 10.1016/j.neuroscience.2010.05.001 20457224

[B58] Matsutani S. Yamamoto N. (2008). Centrifugal innervation of the mammalian olfactory bulb. Anat. Sci. Int. 83, 218–227 10.1111/j.1447-073x.2007.00223.x 19159349

[B59] Mclean J. H. Shipley M. T. (1987). Serotonergic afferents to the rat olfactory bulb: I. Origins and laminar specificity of serotonergic inputs in the adult rat. J. Neurosci. 7, 3016–3028 2822862 10.1523/JNEUROSCI.07-10-03016.1987 PMC6569188

[B60] McLean J. H. Shipley M. T. Nickell W. T. Aston-Jones G. Reyher C. K. (1989). Chemoanatomical organization of the noradrenergic input from locus coeruleus to the olfactory bulb of the adult rat. J. Comp. Neurol. 285, 339–349 10.1002/cne.902850305 2547851

[B61] Meyer E. A. Illig K. R. Brunjes P. C. (2006). Differences in chemo- and cytoarchitectural features within pars principalis of the rat anterior olfactory nucleus suggest functional specialization. J. Comp. Neurol. 498, 786–795 10.1002/cne.21077 16927267 PMC1592518

[B62] Meyer F. Louilot A. (2012). Early prefrontal functional blockade in rats results in schizophrenia-related anomalies in behavior and dopamine. Neuropsychopharmacology 37, 2233–2243 10.1038/npp.2012.74 22588351 PMC3422488

[B63] Miyamichi K. Amat F. Moussavi F. Wang C. Wickersham I. Wall N. R. (2011). Cortical representations of olfactory input by trans-synaptic tracing. Nature 472, 191–196 10.1038/nature09714 21179085 PMC3073090

[B64] Nagayama S. Enerva A. Fletcher M. L. Masurkar A. V. Igarashi K. M. Mori K. (2010). Differential axonal projection of mitral and tufted cells in the mouse main olfactory system. Front. Neural Circuits 4:120 10.3389/fncir.2010.00120 20941380 PMC2952457

[B65] Nagayama S. Takahashi Y. K. Yoshihara Y. Mori K. (2004). Mitral and tufted cells differ in the decoding manner of odor maps in the rat olfactory bulb. J. Neurophysiol. 91, 2532–2540 10.1152/jn.01266.2003 14960563

[B66] Niell C. M. Stryker M. P. (2010). Modulation of visual responses by behavioral state in mouse visual cortex. Neuron 65, 472–479 10.1016/j.neuron.2010.01.033 20188652 PMC3184003

[B67] Nunez-Parra A. Li A. Restrepo D. (2014). Coding odor identity and odor value in awake rodents. Prog. Brain Res. 208, 205–222 10.1016/b978-0-444-63350-7.00008-5 24767484 PMC4131676

[B68] Nunez-Parra A. Maurer R. K. Krahe K. Smith R. S. Araneda R. C. (2013). Disruption of centrifugal inhibition to olfactory bulb granule cells impairs olfactory discrimination. Proc. Natl. Acad. Sci. U S A 110, 14777–14782 10.1073/pnas.1310686110 23959889 PMC3767551

[B69] Ojima H. Yamasaki T. Kojima H. Akashi A. (1988). Cholinergic innervation of the main and the accessory olfactory bulbs of the rat as revealed by a monoclonal antibody against choline acetyltransferase. Anat. Embryol. (Berl) 178, 481–488 10.1007/bf00305035 3223607

[B112] Paxinos G. Franklin K. B. J. (2001). The Mouse Brain in Stereotaxic Coordinates. 2nd Edn San Diego: Academic Press

[B70] Petreanu L. Gutnisky D. A. Huber D. Xu N. L. O’connor D. H. Tian L. (2012). Activity in motor-sensory projections reveals distributed coding in somatosensation. Nature 489, 299–303 10.1038/nature11321 22922646 PMC3443316

[B71] Petrovich G. D. Risold P. Y. Swanson L. W. (1996). Organization of projections from the basomedial nucleus of the amygdala: a PHAL study in the rat. J. Comp. Neurol. 374, 387–420 10.1002/(sici)1096-9861(19961021)374:3<387::aid-cne6>3.0.co;2-y 8906507

[B72] Petzold G. C. Hagiwara A. Murthy V. N. (2009). Serotonergic modulation of odor input to the mammalian olfactory bulb. Nat. Neurosci. 12, 784–791 10.1038/nn.2335 19430472

[B73] Pologruto T. Sabatini B. Svoboda K. (2003). ScanImage: flexible software for operating laser scanning microscopes. Biomed. Eng. Online 2:13 10.1186/1475-925X-2-13 12801419 PMC161784

[B74] Price J. L. Powell T. P. (1970). An experimental study of the origin and the course of the centrifugal fibres to the olfactory bulb in the rat. J. Anat. 107, 215–237 5487119 PMC1234020

[B75] Rabbitt R. D. Brownell W. E. (2011). Efferent modulation of hair cell function. Curr. Opin. Otolaryngol. Head Neck Surg. 19, 376–381 10.1097/MOO.0b013e32834a5be1 22552698 PMC3343276

[B76] Reyher C. K. Schwerdtfeger W. K. Baumgarten H. G. (1988). Interbulbar axonal collateralization and morphology of anterior olfactory nucleus neurons in the rat. Brain Res. Bull. 20, 549–566 10.1016/0361-9230(88)90214-6 2454708

[B77] Rogers S. W. Gahring L. C. (2012). Nicotinic receptor Alpha7 expression during tooth morphogenesis reveals functional pleiotropy. PLoS One 7:e36467 10.1371/journal.pone.0036467 22666322 PMC3364260

[B78] Rogers S. W. Myers E. J. Gahring L. C. (2012a). The expression of nicotinic receptor alpha7 during cochlear development. Brain Behav. 2, 628–639 10.1002/brb3.84 23139908 PMC3489815

[B79] Rogers S. W. Tvrdik P. Capecchi M. R. Gahring L. C. (2012b). Prenatal ablation of nicotinic receptor alpha7 cell lineages produces lumbosacral spina bifida the severity of which is modified by choline and nicotine exposure. Am. J. Med. Genet. A 158A, 1135–1144 10.1002/ajmg.a.35372 22473653 PMC3415211

[B80] Rothermel M. Brunert D. Zabawa C. Díaz-Quesada M. Wachowiak M. (2013). Transgene expression in target-defined neuron populations mediated by retrograde infection with adeno-associated viral vectors. J. Neurosci. 33, 15195–15206 10.1523/JNEUROSCI.1618-13.2013 24048849 PMC3776063

[B81] Rothermel M. Carey R. M. Puche A. Shipley M. T. Wachowiak M. (2014). Cholinergic inputs from basal forebrain add an excitatory bias to odor coding in the olfactory bulb. J. Neurosci. 34, 4654–4664 10.1523/JNEUROSCI.5026-13.2014 24672011 PMC3965788

[B82] Schneider S. P. Scott J. W. (1983). Orthodromic response properties of rat olfactory bulb mitral and tufted cells correlate with their projection patterns. J. Neurophysiol. 50, 358–378 6886740 10.1152/jn.1983.50.2.358

[B83] Schoenfeld T. A. Macrides F. (1984). Topographic organization of connections between the main olfactory bulb and pars externa of the anterior olfactory nucleus in the hamster. J. Comp. Neurol. 227, 121–135 10.1002/cne.902270113 6470206

[B84] Schoenfeld T. A. Marchand J. E. Macrides F. (1985). Topographic organization of tufted cell axonal projections in the hamster main olfactory bulb: an intrabulbar associational system. J. Comp. Neurol. 235, 503–518 10.1002/cne.902350408 2582006

[B85] Scott J. W. (1981). Electrophysiological identification of mitral and tufted cells and distributions of their axons in olfactory system of the rat. J. Neurophysiol. 46, 918–931 6271931 10.1152/jn.1981.46.5.918

[B86] Scott J. W. Mcbride R. L. Schneider S. P. (1980). The organization of projections from the olfactory bulb to the piriform cortex and olfactory tubercle in the rat. J. Comp. Neurol. 194, 519–534 10.1002/cne.901940304 7451680

[B87] Scott J. W. Ranier E. C. Pemberton J. L. Orona E. Mouradian L. E. (1985). Pattern of rat olfactory bulb mitral and tufted cell connections to the anterior olfactory nucleus pars externa. J. Comp. Neurol. 242, 415–424 10.1002/cne.902420309 4086669

[B88] Shea S. D. Katz L. C. Mooney R. (2008). Noradrenergic induction of odor-specific neural habituation and olfactory memories. J. Neurosci. 28, 10711–10719 10.1523/JNEUROSCI.3853-08.2008 18923046 PMC2588668

[B89] Shipley M. T. Adamek G. D. (1984). The connections of the mouse olfactory bulb: a study using orthograde and retrograde transport of wheat germ agglutinin conjugated to horseradish peroxidase. Brain Res. Bull. 12, 669–688 10.1016/0361-9230(84)90148-5 6206930

[B90] Shipley M. T. Halloran F. J. de la Torre J. (1985). Surprisingly rich projection from locus coeruleus to the olfactory bulb in the rat. Brain Res. 329, 294–299 10.1016/0006-8993(85)90537-2 3978450

[B91] Shusterman R. Smear M. C. Koulakov A. A. Rinberg D. (2011). Precise olfactory responses tile the sniff cycle. Nat. Neurosci. 14, 1039–1044 10.1038/nn.2877 21765422 PMC13348895

[B92] Soria-Gómez E. Bellocchio L. Reguero L. Lepousez G. Martin C. Bendahmane M. (2014). The endocannabinoid system controls food intake via olfactory processes. Nat. Neurosci. 17, 407–415 10.1038/nn.3647 24509429

[B93] Sosulski D. L. Lissitsyna Bloom M. Cutforth T. Axel R. Datta S. R. (2011). Distinct representations of olfactory information in different cortical centres. Nature 472, 213–216 10.1038/nature09868 21451525 PMC3354569

[B94] Soucy E. R. Albeanu D. F. Fantana A. L. Murthy V. N. Meister M. (2009). Precision and diversity in an odor map on the olfactory bulb. Nat. Neurosci. 12, 210–220 10.1038/nn.2262 19151709

[B95] Spors H. Wachowiak M. Cohen L. B. Friedrich R. W. (2006). Temporal dynamics and latency patterns of receptor neuron input to the olfactory bulb. J. Neurosci. 26, 1247–1259 10.1523/jneurosci.3100-05.2006 16436612 PMC6674558

[B96] Stratford T. R. Wirtshafter D. (2012). Effects of muscimol, amphetamine and DAMGO injected into the nucleus accumbens shell on food-reinforced lever pressing by undeprived rats. Pharmacol. Biochem. Behav. 101, 499–503 10.1016/j.pbb.2012.02.010 22366216 PMC3310292

[B97] Strowbridge B. W. (2009). Role of cortical feedback in regulating inhibitory microcircuits. Ann. N Y Acad. Sci. 1170, 270–274 10.1111/j.1749-6632.2009.04018.x 19686146

[B98] Swanson L. W. Cowan W. M. (1977). An autoradiographic study of the organization of the efferent connections of the hippocampal formation in the rat. J. Comp. Neurol. 172, 49–84 10.1002/cne.901720104 65364

[B99] Tian L. Hires S. A. Mao T. Huber D. Chiappe M. E. Chalasani S. H. (2009). Imaging neural activity in worms, flies and mice with improved GCaMP calcium indicators. Nat. Methods 6, 875–881 10.1038/nmeth.1398 19898485 PMC2858873

[B100] Valverde F. Lopez-Mascaraque L. De Carlos J. A. (1989). Structure of the nucleus olfactorius anterior of the hedgehog (Erinaceus europaeus). J. Comp. Neurol. 279, 581–600 10.1002/cne.902790407 2465323

[B101] van Groen T. Wyss J. M. (1990). Extrinsic projections from area CA1 of the rat hippocampus: olfactory, cortical, subcortical and bilateral hippocampal formation projections. J. Comp. Neurol. 302, 515–528 10.1002/cne.903020308 1702115

[B102] Verhagen J. V. Wesson D. W. Netoff T. I. White J. A. Wachowiak M. (2007). Sniffing controls an adaptive filter of sensory input to the olfactory bulb. Nat. Neurosci. 10, 631–639 10.1038/nn1892 17450136

[B103] Wachowiak M. Cohen L. B. (2001). Representation of odorants by receptor neuron input to the mouse olfactory bulb. Neuron 32, 723–735 10.1016/s0896-6273(01)00506-2 11719211

[B104] Wachowiak M. Economo M. N. Díaz-Quesada M. Brunert D. Wesson D. W. White J. A. (2013). Optical dissection of odor information processing in vivo using GCaMPs expressed in specified cell types of the olfactory bulb. J. Neurosci. 33, 5285–5300 10.1523/JNEUROSCI.4824-12.2013 23516293 PMC3690468

[B105] Weeg M. S. Land B. R. Bass A. H. (2005). Vocal pathways modulate efferent neurons to the inner ear and lateral line. J. Neurosci. 25, 5967–5974 10.1523/jneurosci.0019-05.2005 15976085 PMC6724790

[B106] Wesson D. W. Carey R. M. Verhagen J. V. Wachowiak M. (2008). Rapid encoding and perception of novel odors in the rat. PLoS Biol. 6:e82 10.1371/journal.pbio.0060082 18399719 PMC2288628

[B107] Woolf N. J. Eckenstein F. Butcher L. L. (1984). Cholinergic systems in the rat brain: I. projections to the limbic telencephalon. Brain Res. Bull. 13, 751–784 10.1016/0361-9230(84)90236-3 6532518

[B108] Yan Z. Tan J. Qin C. Lu Y. Ding C. Luo M. (2008). Precise circuitry links bilaterally symmetric olfactory maps. Neuron 58, 613–624 10.1016/j.neuron.2008.03.012 18498741

[B109] Zaborszky L. Van Den Pol A. N. Gyengesi E. (2012). “The basal forebrain cholinergic projection system in mice,” in The Mouse Nervous System, eds Watson C. Paxinos G. Puelles L. (Amsterdam: Elsevier), 684–718

[B110] Zariwala H. A. Borghuis B. G. Hoogland T. M. Madisen L. Tian L. De Zeeuw C. I. (2012). A cre-dependent GCaMP3 reporter mouse for neuronal imaging in vivo. J. Neurosci. 32, 3131–3141 10.1523/JNEUROSCI.4469-11.2012 22378886 PMC3315707

[B111] Zhuravin I. A. Bures J. (1991). Extent of the tetrodotoxin induced blockade examined by pupillary paralysis elicited by intracerebral injection of the drug. Exp. Brain Res. 83, 687–690 10.1007/bf00229849 2026211

